# Complete chloroplast genome sequence of *Populus euphratica* from PacBio Sequel platform

**DOI:** 10.1080/23802359.2020.1869605

**Published:** 2021-02-08

**Authors:** Zhen-Bo Jiang, Jun-Tuan Zhai, Pei-Pei Jiao, Tian-Ge Yang, Zhi-Hua Wu, Zhi-Jun Li

**Affiliations:** a Xinjiang Production & Construction Corps Key Laboratory of Protection and Utilization of Biological Resources in Tarim Basin, Tarim University, Alar, PR China; b Desert Poplar Research Center, Tarim University, Alar, PR China; c College of Life Science, Tarim University, Alar, PR China; d Hubei Provincial Key Laboratory for Protection and Application of Special Plant Germplasm in Wuling Area of China & Key Laboratory of State Ethnic Affairs Commission for Biological Technology, College of Life Sciences, South-Central University for Nationalities, Wuhan, PR China

**Keywords:** *Populus euphratica*, chloroplast genome, PacBio Sequel, evolution

## Abstract

*Populus euphratica* Oliv., one of tall arbors growing in desert areas, has great stress resistance. The complete chloroplast genome was reported in this study using the PacBio Sequel Platform. The chloroplast genome with a total size of 157,881 bp consisted of two inverted repeats (IRs) (27,666 bp) separated by a large single-copy region (85,906 bp) and a small single-copy region (16,643 bp). Further annotation revealed the chloroplast genome contains 111 genes, including 77 protein-coding genes, 30 *tRNA* genes, and four *rRNA* genes. The information of the chloroplast genome will be useful for study on the evolution of *P. euphratica* in the future.

*Populus euphratica* Oliv., is the natural arbor species that can survive in the serious desert environments and exhibits remarkable resistance to environmental stresses (Lv et al. 2014). Due to its greater ability to cope with environmental stresses, *P. euphratica* is widely considered as an ideal model system when studying the molecular mechanisms of abiotic stress responses in woody species (Sun et al. 2009; Ding et al. 2010). In this study, to obtain the new insight into the evolution of *P. euphratica*, we sequenced, assembled, and annotated the accurate chloroplast genome with PacBio Sequel platform.

The materials of *P. euphratica* in this study were collected from *P. euphratica* forest in the headwater region of the Tarim River on the northwestern margin of the Tarim basin in Xinjiang province of China (81°17′56.52′′E, 40°32′36.90′′N, 980 m above sea level). The voucher specimens were deposited at the Herbarium of Tarim University (TD-00301). The leaves total genomic DNA was extracted using a modified cetyltrimethylammonium bromide (CTAB) method and sequenced using the PacBio platform. The raw sequencing data (SRR12959747) generated 35,960 reads with the N50 of 10,213 bp. The whole Chloroplast genomes were assembled from whole genome sequencing data using Canu (Koren et al. 2017) and got 15 contigs with the N50 of 21,246 bp. To discard nuclear DNA sequences and obtain the complete chloroplast genome sequence, we aligned the contigs of a preliminary assembly to the whole chloroplast data from NCBI. Then the draft genome was polished with Arrow (SMRT link-6.0.0, Pacific Biosciences, Menlo Park, CA). Due to the special structure of the chloroplast genome, we mapped the scaffolds to the reference to find the IR region and manually adjusted. Then annotated using CPGAVAS2 (Shi et al. 2019) and PGA (Qu et al. 2019). The complete chloroplast genome was 157,881 bp (MT818237) and composed of two inverted repeats (IRs) of 27,666 bp each, which divide a large single copy (LSC) region of 85,906 bp and a small single copy (SSC) region of 16,643 bp, the average GC content was 36.53%. The chloroplast genomes encoded 111 genes, including 77 protein-coding genes, 30 *tRN*A genes, and four *rRNA* genes.

According to the previously published chloroplast genome of *P. euphratica* from NCBI with Illumina platform (NC_024747), we aligned the *P. euphratica* chloroplast of Illumina and PacBio platforms using BLASTN. PacBio RS data can produce high-quality sequence assemblies covering a greater proportion of the genome than can be achieved by Illumina sequencing alone. We found that the cp genome got from PacBio platform was slightly longer. After designing the primers (5′- AATGTAGGATTAGCGGTTCT-3′′ and 5′-GCTGTATTCATGCCTGTTCG-3′′,5′-TAACCTGCTCTGTCTGGACT-3′′, and 5′-CTTGTACTTGCTGCTTGCTT-3′′) for different places between the genome with two platforms, we verified the real existence of the insertion assembled by PacBio through Sanger. The result showed that the PacBio has the advantage of getting more complete chloroplast genome, which is also reported in other plants (Wu et al. 2014).

In our study, to explore the phylogenetic relationship of *P. euphratica* within Salicaeae, additional 25 species from Salicaeae were studied. With the species of *Ricinus communis* L. as the outgroup, the phylogenetic trees were built from the whole protein-coding gene matrix by maximum-likelihood (ML) and Bayesian inference (BI) (Figure 1). The ML tree was generated using IQ-TREE (Nguyen et al. 2015) based on the best model of TVM + F+R3 and 1000 bootstrap replicates, and BI analysis was performed in MrBayes version 3.2.7(Ronquist et al. 2012). This result showed that the *P. euphratica* was closer to the species of *P. pruinosa.*

**Figure 1.**
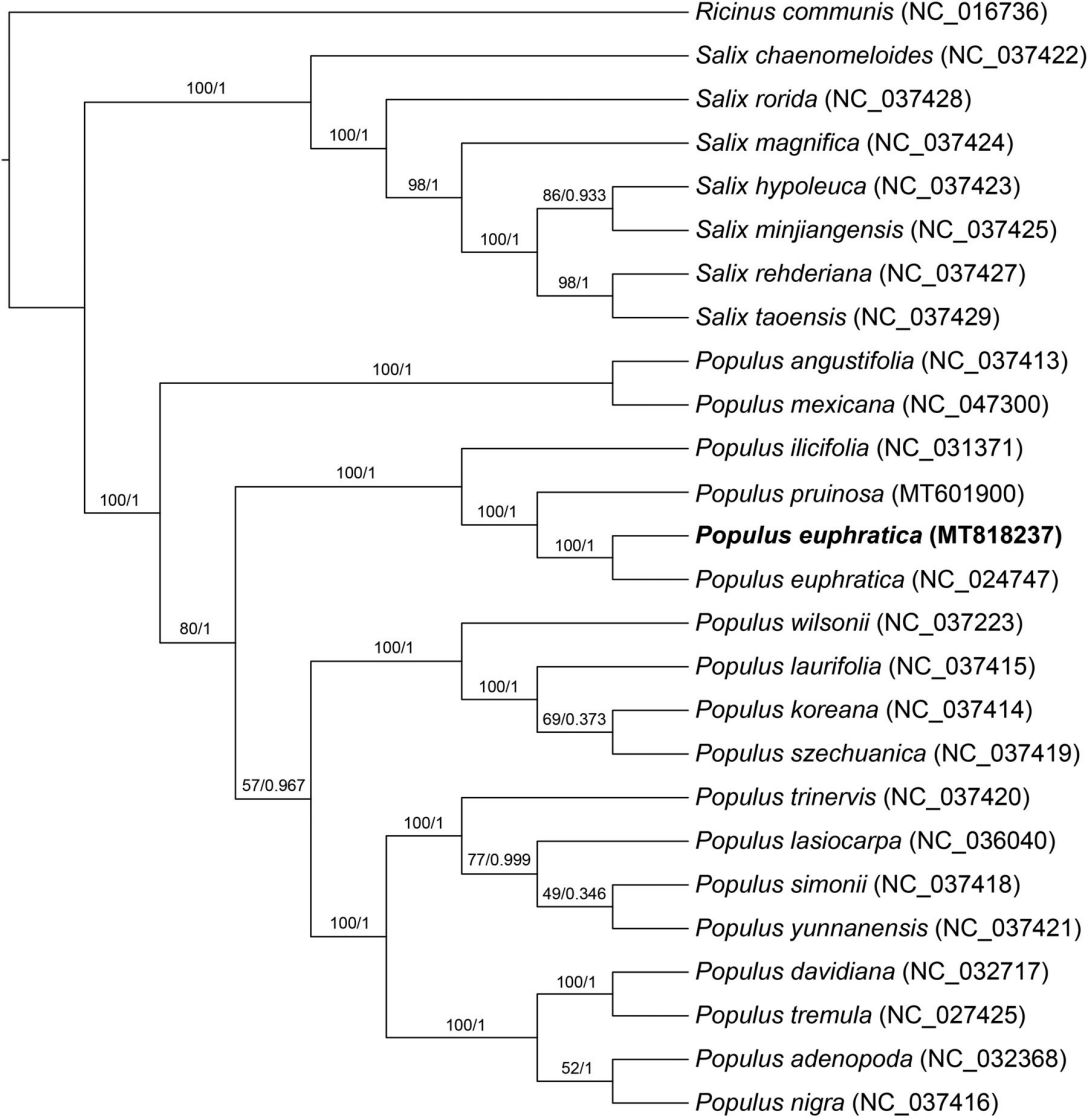
Phylogenetic tree reconstructed by maximum-likelihood (ML) and Bayesian inference (BI) analysis based on the whole chloroplast protein-coding genes of these 26 species.

## Data Availability

The genome sequence data that support the findings of this study are openly available in GenBank of NCBI at [https://www.ncbi.nlm.nih.gov] under the accession no. MT818237. The associated ‘BioProject’, ‘SRA’, and ‘Bio-Sample’ numbers are PRJNA673650, SRR12959747, and SAMN16619580, respectively.
